# Trends among platelet function, arterial calcium, and vascular function measures

**DOI:** 10.1080/09537104.2023.2238835

**Published:** 2023-12

**Authors:** Jason Cunha, Melissa V. Chan, Bongani B. Nkambule, Florian Thibord, Amber Lachapelle, Robin E. Pashek, Ramachandran S. Vasan, Jian Rong, Emelia J. Benjamin, Naomi M. Hamburg, Ming-Huei Chen, Gary F. Mitchell, Andrew D. Johnson

**Affiliations:** 1 National Heart, Lung and Blood Institute’s the Framingham Heart Study, Boston University and National Heart, Framingham, MA, USA; 2 National Heart, Lung and Blood Institute, Population Sciences Branch, Framingham, MA, USA; 3 Cardiology and Preventive Medicine Sections, Department of Medicine, Boston University Chobanian and Avedisian School of Medicine, Boston, MA, USA; 4 Department of Epidemiology, Boston University School of Public Health, Boston, MA, USA; 5 Evans Center for Interdisciplinary Biomedical Research, Boston University Chobanian and Avedisian School of Medicine, Boston, MA, USA; 6 Whitaker Cardiovascular Institute, Boston University Chobanian and Avedisian School of Medicine, Boston, MA, USA; 7 Schools of Public Health and Medicine, Departments of Population Health and Medicine, University of Texas Health Sciences Center, San Antonio, TX, USA; 8 Department of Biostatistics, Boston University School of Public Health, Boston, MA, USA; 9 Cardiovascular Engineering, Inc, Norwood, MA, USA

**Keywords:** Aortic diameter, arterial tonometry, epidemiology, Platelet, vascular calcification

## Abstract

Arterial tonometry and vascular calcification measures are useful in cardiovascular disease (CVD) risk assessment. Prior studies found associations between tonometry measures, arterial calcium, and CVD risk. Activated platelets release angiopoietin-1 and other factors, which may connect vascular structure and platelet function. We analyzed arterial tonometry, platelet function, aortic, thoracic and coronary calcium, and thoracic and abdominal aorta diameters measured in the Framingham Heart Study Gen3/NOS/OMNI-2 cohorts (*n* = 3,429, 53.7% women, mean age 54.4 years ±9.3). Platelet reactivity in whole blood or platelet-rich plasma was assessed using 5 assays and 7 agonists. We analyzed linear mixed effects models with platelet reactivity phenotypes as outcomes, adjusting for CVD risk factors and family structure. Higher arterial calcium trended with higher platelet reactivity, whereas larger aortic diameters trended with lower platelet reactivity. Characteristic impedance (Zc) and central pulse pressure positively trended with various platelet traits, while pulse wave velocity and Zc negatively trended with collagen, ADP, and epinephrine traits. All results did not pass a stringent multiple test correction threshold (*p* < 2.22e-04). The diameter trends were consistent with lower shear environments invoking less platelet reactivity. The vessel calcium trends were consistent with subclinical atherosclerosis and platelet activation being inter-related.

## Introduction

Platelet reactivity measures can be useful in cardiovascular disease (CVD) risk determination. For example, Berger et al. found that adenosine diphosphate (ADP) and trimethylamine N-oxide-induced platelet reactivity were significant independent predictors of cardiovascular and all-cause mortality in the LUdwigshafen RIsk and Cardiovascular Health (LURIC) Study.^1^ Measures such as increased platelet count, volume, and reactivity have been correlated with increased risk for acute myocardial infarction and poor cardiovascular outcomes.^1–4^ Platelets have been shown to release angiopoietin-1, endothelial, and transforming growth factors, which decrease endothelial permeability to plasma under normal circumstances.^5,6^ In cases of endothelial barrier breaks and impairments, the exposed collagen layer that normally lies beneath the endothelium promotes platelet binding to the injury site, allowing clots to form.^7^ When this occurs, platelets can release endothelial cell adhesion molecules, such as P-selectin and recruit leukocytes, facilitating the inflammatory response.^8,9^ In addition, von Willebrand factor (vWF) generated from platelet alpha-granules and endothelial cells helps initiate platelet–platelet adhesion and binding to sites of injury along the vascular wall, aiding in thrombus formation.^10^ Under shear stress conditions, platelets can release microparticles with various pro-coagulant factors, including exposure of phosphatidylserine and glycoproteins (GPs) like GPIIb/IIIa and GPIb/IX.^11–13^ Hence, platelets have an important influence on hemostasis and vascular function under a variety of conditions in the human body.

One way to measure arterial function is through tonometry methods such as carotid-femoral pulse wave velocity (CFPWV), which is a measure of aortic wall stiffness.^14,15^ In conjunction with computed tomography (CT) scans for coronary artery calcium (CAC),^16^ endothelial function and tonometry data may aid in CVD risk determination.^17–19^ In one study, Zachariah et al. found positive associations between serum vascular endothelial growth factors like angiopoietin-2, CFPWV, and mean arterial pressure, as well as negative associations between insulin-like growth factor 1 concentrations, CFPWV, and mean arterial pressure.^20^ This community-based cross-sectional study indicated that vascular growth factor levels were related to measures of mean and pulsatile hemodynamic load.^20^

Few studies have effectively assessed the associations between platelet activation markers, arterial calcium, vessel diameter, and tonometry measures. Other researchers have primarily analyzed data gathered from high-resolution ultrasound and peripheral artery tonometry to measure vascular functioning, all while not factoring in direct measures of platelet function or arterial bed calcium burden measurements.^21,22^ Studies that have investigated the connections between tonometry measures, either direct or indirect platelet activation markers, and cardiovascular outcomes under various medical conditions and treatment regimens tended to have sample sizes of approximately 50 or fewer individuals, which may lack statistical power and generalizability.^23–27^ Given that various platelet,^1–4^ tonometry,^14,15^ diameter,^28,29^ and calcium^16^ measures were found to be independently associated with CVD outcomes, it may be possible that the latter three sets of traits have an influence on platelet reactivity, and vice versa. In this study, we sought to unify these quantitative measurement types in a single large cohort with comprehensive platelet phenotyping to address whether platelet reactivity is interrelated to other vessel and CVD-associated measures.

Here, we examine relations between platelet function, arterial tonometry, arterial calcium CT scans, and aortic and arterial diameter measures in the large, community-based Framingham Heart Study (FHS) Generation 3, New Offspring Spouse (NOS), and diverse OMNI-2 cohorts of middle-aged and older adult men and women. Due to the previous independent associations between platelet, arterial tonometry, diameter, and calcium measures, we hypothesized that higher arterial wall calcium burden and higher arterial stiffness are related to higher platelet aggregation measures, whereas higher aortic diameter measures are negatively related to platelet aggregation.

## Methods and materials

### Cohort information

This study included participants from the FHS Generation 3, NOS, and OMNI-2 cohorts, which have been previously described.^30–32^ Participants return to the FHS clinic with gaps of several years to undergo examinations for various CVD risk factors and physical examinations. This research protocol was approved by the FHS and the Boston University Institutional Review Board. Informed consent was obtained from participants on the day of their examinations. Figure S1 provides timelines of when each phenotype was measured during their respective Exam periods. Platelet and tonometry measures were gathered from Gen3/NOS/OMNI-2 cohorts during Exam 3 (2016–2019), vessel calcium measures were gathered from Gen3/OMNI-2 cohorts during Exam 2 (2008–2011), and vessel diameter measures were gathered from Gen3 cohort during Exam 1 (2002–2005). Diameter and calcium data were not available for the NOS cohort, and diameter, CAC, and AAS data were not available for the OMNI-2 cohort. There were large discrepancies in the calcium trait sample sizes because ~50% of participants did not participate in the imaging call-back examination.^33^ There were also exclusions applied for CT measures based on age (<35 years of age for men or <40 years of age for women), weight (≥160 kg), and pregnancy;^33^ those individuals with imaging measures had higher mean BMI, diabetes rates, and medication use at the time of the platelet function exam (Exam 3). Demographic differences between those with and without vessel imaging measures are included in Table S2.

Physical examinations were used to gather CVD risk factor measures such as blood pressure, and laboratory evaluations of participant blood samples were conducted. Enzymatic methods were used to measure plasma cholesterol and triglyceride concentrations, and high-density lipoprotein cholesterol (HDL-C) was measured after using dextran sulfate-magnesium precipitation of apolipoprotein B-containing lipoproteins. Blood glucose measurements were done using a hexokinase reagent. We included all participants with available data on arterial tonometry, aortic diameter, arterial wall calcium scores, and platelet function parameters.

### Blood collection

Fasting blood was collected from research participants via venipuncture of the antecubital vein in a supine position, and either platelet-rich plasma (PRP) or whole blood (WB) were used in platelet function assays. Phlebotomists used a 21 gauge safety lock blood collection system (BD Biosciences; San Jose, CA).^34^

We used two hirudin blood tubes (3.0 mL; Diapharma; Westchester, OH) for WB assays. These tubes rested at room temperature for 30 minutes prior to platelet testing. In addition, three 4.5 mL sodium citrate (3.2%) tubes (BD; San Jose, CA) were collected for PRP assays.^34^

### Platelet-rich plasma isolation

We centrifuged the sodium citrate tubes at 200 × g for 10 minutes with no brake to isolate PRP (Sorvall ST8 centrifuge, Thermo Sci 75 003 181 rotor), and 1500 × g for 15 minutes with no brake to isolate platelet-poor plasma. Sodium citrate tubes were rested at room temperature for 15 minutes before centrifugation. These preparations were made in accordance with the International Society on Thrombosis and Hemostasis guidelines.^34^

### Platelet reactivity measurements

**Figure 1.**
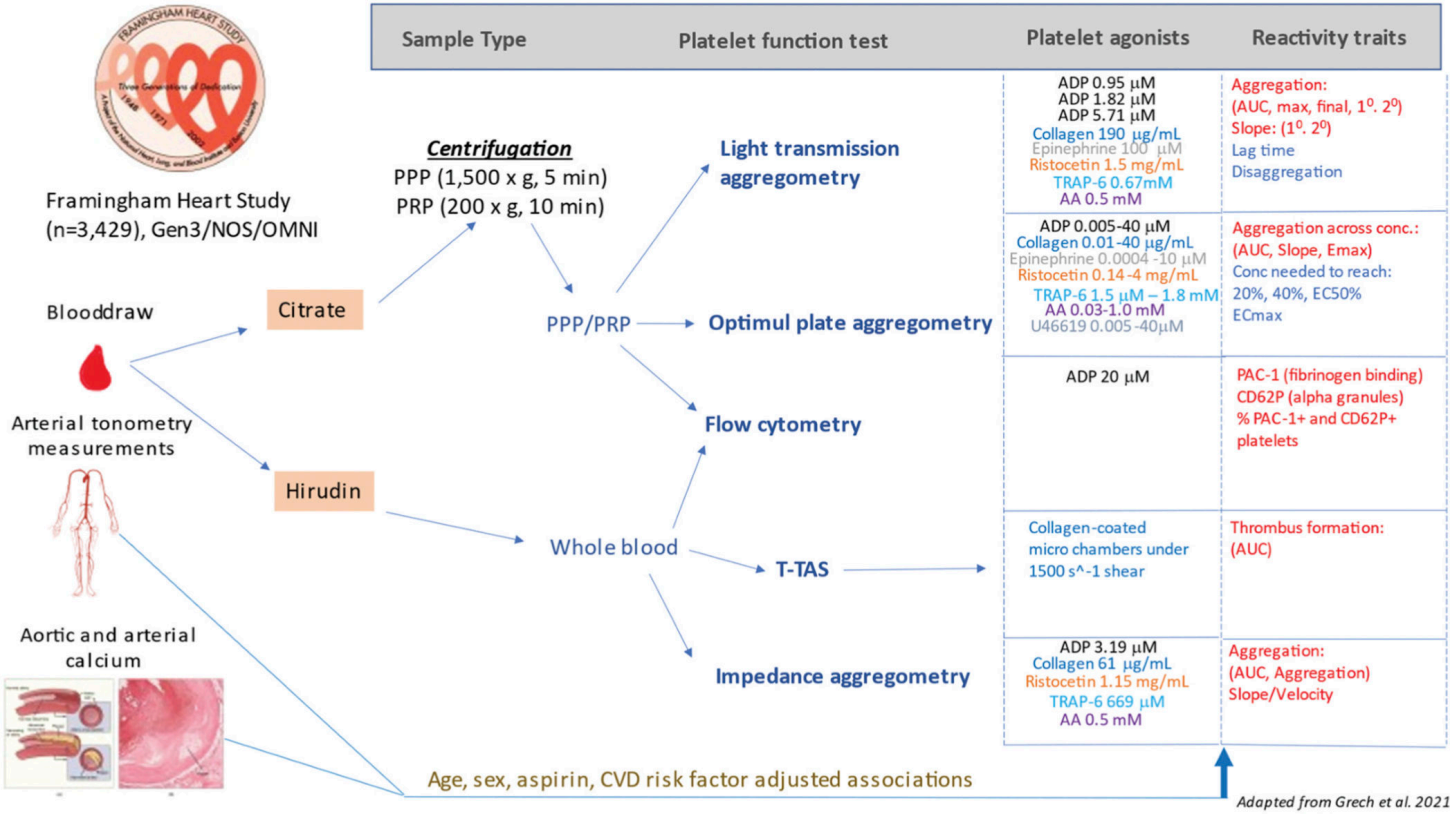
Overview of study design (adapted from Grech et al. 2021). Whole blood was drawn from OMNI-2, Gen3, and NOS participants during Exam 3 (2016–2019). Whole blood and isolated PRP were used to take platelet activation measures using 5 different assays. Arterial tonometry measures were also gathered from these cohorts during Exam 3. Calcium measures were gathered from Gen3/OMNI-2 participants during Exam 2 (2008–2011), and CT vessel diameters were gathered from Gen3 participants during Exam 1 (2002–2005). A linear mixed effect model, which adjusted for age, sex, aspirin, and various CVD risk factors was used to assess the associations between the three main variable categories. Image sources: Arterial tonometry image: by Mikael Häggström, MD. Public Domain (CC0 1.0). Aortic and arterial calcium image: This Photo by Unknown Author is licensed under CC BY-SA.

We utilized a variety of assays to measure different platelet reactivity traits in both WB and PRP samples when exposed to different agonists. These measurements were recently described in detail.^34^ They are summarized below, and a more detailed list of assay components and platelet agonist concentrations is found in Figure 1. Agonists used for these platelet reactivity assays were acquired in large lots from Bio/ Data (Horsham, PA) and Roche Diagnostics (Mannheim, Germany) to reduce batch variability. Assay platform details, platelet reactivity variable definitions, and agonist concentrations are summarized in Table S1.

### Whole blood impedance aggregometry

We utilized a 5-channel Multiplate analyzer (Roche Diagnostics; Mannheim, Germany) to collect whole blood impedance aggregometry traits, including area-under-the-curve (AUC), velocity, and aggregation for each agonist. The platelet agonists used for this assay were 0.22 mM thrombin receptor activator peptide-6 (TRAP-6), 3.19 μM ADP, 1.15 mg/mL ristocetin, 0.5 mM arachidonic acid (AA), and 61 μg/mL collagen.

### Shear-stress mediated platelet thrombus formation

We used the Total-Thrombus Formation Assay System (T-TAS) Plus (Zacros; Tokyo, Japan) to assess shear stress-mediated platelet occlusion from whole blood samples when exposed to collagen. Measurements were made under the “PL chip” and “mid- shear” 1500 1/s settings. T-TAS AUC was analyzed as a platelet trait.

### Light transmission aggregometry (LTA)

We used an eight-channel PAP-8E light transmission aggregometer (Bio/Data; Horsham, PA, USA) to collect aggregation measures from PRP. Measurements were made in accordance with the International Society on Thrombosis and Hemostasis guidelines.^35^ The agonists used were 0.95 μM, 1.82 μM, and 5.71 μM ADP, 190 μg/mL collagen, 1.6 mM AA, 100 μM epinephrine, 0.67 mM TRAP-6, and 1.5 mg/mL ristocetin. AUC, percent final aggregation, percent maximal aggregation, primary aggregation, and primary slope were collected for each agonist. Lag time for AA and collagen, secondary aggregation and secondary slope for ADP, and epinephrine, and disaggregation to ADP and ristocetin were also calculated. The AUC provided by the instrument corresponds to the sum of the average aggregation percentage measured each second, divided by 60 seconds. This method could induce bias if samples were not processed for the same duration, resulting in potentially inaccurate comparisons between samples. To address this, we recomputed the AUC from the aggregation percentages, rescaled between 0 and 1, and divided the result by the total duration of the assay after agonist injection, in seconds (in most cases 360 seconds). This resulted in a standardized AUC with values ranging from 0 to 1.

### Optimul 96-well plate assay

We used the Optimul 96-well plate assay to assess platelet reactivity to varying concentrations of the following platelet agonists: 0.03–1.0 mM AA, 0.005–40 μM ADP, 0.01–40 μg/mL collagen, 0.0004–10 μM epinephrine, 0.14–4.0 mg/mL ristocetin, 1.5 μM– 1.8 mM TRAP-6, and 0.005–40 μM U46619.^34,36^ The plates used for the assay were made by members of our research group in Framingham, MA, USA. Optimul AUC mean, ec50, eMax, concentration to reach 20% (Agg20) and 40% aggregation (Agg40), ecMax, and slope were collected for each agonist. These calculations were done using the NPLR package.^37^

### Flow cytometry data preparation, cleaning, and analysis

Flow cytometry data was acquired using the BD Accuri C6 instrument, and the BD Accuri analysis software (BD Biosciences; San Jose, CA, USA) was used for gating and export of data for analysis by linear mixed effects models in R. Flow cytometry traits, all of which measured in both PRP and WB, include percent positive PAC-1 and P-selectin platelets; and normal saline estimated platelet count. We prepared a 1:40 dilution of both PRP and WB by adding 10 μL of PRP and WB, respectively, to 390 μL of phosphate buffered solution. 45 μL of each dilution mix was added to 5 μL of normal saline and 5 μL of 200 μM ADP, respectively, and incubated at 37°C for 15 minutes. Following incubation, 50 μL of antibody cocktail containing 1:20 anti-CD61 antibody (PerCP-Cy5.5 Mouse Anti-Human CD61 Clone VI-PL2), 1:5 anti-CD62P antibody (APC Mouse Anti-Human CD62P; Clone AK-4), and 1:5 anti-PAC-1 antibody (FITC Mouse Anti-Human PAC-1; Clone PAC-1) was added to each tube and incubated at 37°C for 15 minutes. Finally, 1150 μL of staining mix containing three parts stain buffer and one part cytofix solution (BD Biosciences; San Jose, CA, USA) was added to complete the sample preparations.

Each sample was run on a “slow” flow-rate and a primary stop condition set at “10,000 CD61+ events.” Gates were drawn based on experiments with different antibody combination and stimulated or unstimulated conditions, and were cross-checked across random samples throughout every month of the exam period and fine-tuned before developing a final analysis template. To minimize the impact of abnormal events during data acquisition and to exclude events affected by technical artifacts, we performed quality control on all compensated Flow Cytometric Standard (fcs) files using the Flow AI package (version 1.12.7) on FlowJo (version 10.7.1).^38^ The FlowAI package allows for the screening, selection, and exclusion of bad-quality events, based on the evaluation of flow rate abnormalities, parameter instability and omission of fluorescence signals or events that fall out of the specific dynamic range of each fluorescence channel.^38^

### Arterial tonometry and diameter measurements

Participants underwent applanation tonometry in a supine position after 5 minutes of rest as described previously.^39,40^ Systolic and diastolic blood pressure measures were taken in the right arm using a semiautomated auscultatory method (Cardiovascular Engineering Inc, Norwood, MA).^40^ A caliper was used to measure the distance from the suprasternal notch to the femoral site and a tape measure to measure from the suprasternal notch to other sites.^40^

Carotid-femoral pulse wave velocity (CFPWV) was calculated by dividing the carotid-femoral transit distance by the pulse transit time difference between carotid and femoral sites. The difference between transit distances from the suprasternal notch to femoral and carotid sites was used to account for parallel transmission in the brachiocephalic artery and the aortic arch. Raw CFPWV was inverted (1/CFPWV) to limit heteroscedasticity and skewness, then multiplied by −1000 to convert units to ms/m and restore directionality of relations, yielding niCFPWV. Higher values of niCFPWV correspond to higher aortic stiffness.^40^ Central pulse pressure (CPP) was calculated as the difference between carotid systolic and diastolic pressure. Characteristic impedance (Zc), the impedance to pulsatile flow during systole, was calculated in the time domain by dividing the change in pressure by the change in flow in the interval between the flow onset and 95% of peak flow.^41^

The three diameter variables were as follows: ascending aorta (AAo), descending thoracic aorta (DAo), and abdominal aorta (below renal arteries; AbAo). Each diameter variable was the average of orthogonal anteroposterior and mediolateral diameters measured at each respective position.

Primary stiffness measurements included niCFPWV. Secondary stiffness measurements included Zc and central pulse pressure (CPP).

### CT imaging

The cardiac CT examination methods have been previously described.^33^ In summary, CT exams were performed with an eight-slice MDCT scanner (LightSpeed Ultra, General Electric, Milwaukee, WI).^33^

### Image segmentation and traditional Agatston score

Coronary artery scans were segmented into eight segments, and experienced readers labeled all CT scans for both the presence and amount of CAC using open-source software (3DSlicer, v.4.7.0). Agatston scores were multiplied by the area of each lesion along with a weighted attenuation score dependent on the maximal attenuation within the lesion to define the CAC amount.^33,42^

The following scores were calculated: coronary artery calcium (CAC), abdominal aorta calcium (AAS), and thoracic aorta calcium (TAC; average of ascending and descending thoracic aorta diameters). The scores were then dichotomized, such that positive scores had value of 1, due to many 0 values in the three scores (>54%).

### Statistical analyses

Baseline characteristics are shown in Table I. In brief, CVD risk factors, clinical, and demographic information were gathered from individuals in the NOS, Gen3, and OMNI-2 cohorts at Exam 3. Data gathered from a total of 3,429 participants who minimally had platelet function at Exam 3 and tonometry, vessel calcium, or vessel diameter data were included in this study. Overall, the 3,429 FHS participants had a mean age of 54.4 ± 9.3 years, 53.7% were women, and overall had moderate burden of cardiovascular risk factors. The population had moderate prescription rates for anti-lipid treatment (25.2%), anti-hypertensive treatment (28.1%), and based on sensitive AA LTA platelet function assays, 21.3% of individuals had taken recent aspirin and 0.95% of individuals reported taking P2Y12 inhibitors. Platelet and tonometry data were available for all participants included in this study from Exam 3. Demographics of the group with these phenotypes are shown in Table I.

**Figure 2.**
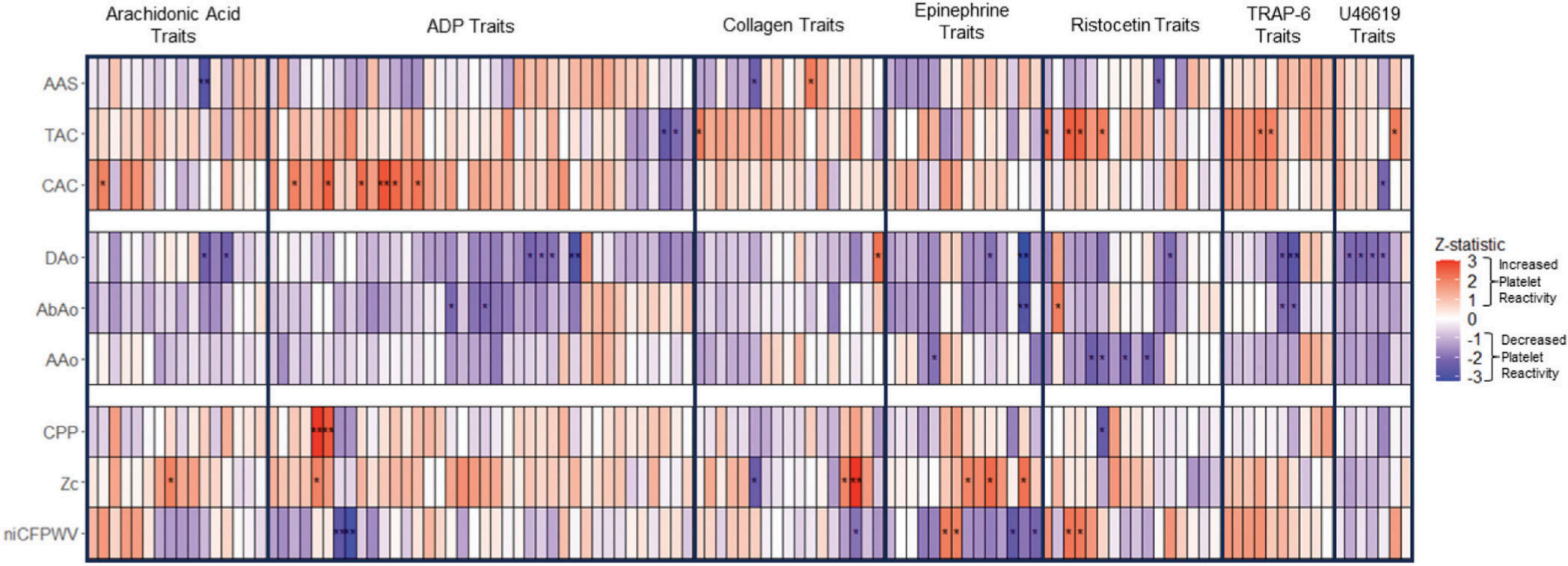
Heatmap of Z statistics associating platelet activation, arterial calcium, tonometry, and diameter measures. Red indicates increased platelet reactivity with an increase in the cardiovascular traits; blue indicates reduced platelet reactivity with a decrease in the cardiovascular traits. * (*p* ≤ .05); ** (*p* ≤ .01). We used linear mixed effects models, which included CVD risk factors, to calculate the betas (β) and standard errors (SE) for this display. Calcium values had 1 added and were ln-transformed. Following this transformation, the β and SE values were used to calculate z-statistics (β/SE). The following z-statistic values were multiplied by −1 to accurately depict the platelet activation directionality: Agg20, Agg40, ecMax, EC50, disaggregation traits (4 total), and lag time traits (2 total). Abbreviations: AAo: antero-posterior diameter of the ascending thoracic aorta obtained at the level of the right pulmonary artery; AAS: abdominal aorta calcium score; AbAo: antero-posterior diameter of the abdominal aorta 5 cm above the aorto-common iliac bifurcation; CAC: coronary artery calcium score; CPP: central pulse pressure; DAo: Antero-posterior diameter of the descending thoracic aorta obtained at the level of the right pulmonary artery; niCFPWV: negative inverse carotid femoral pulse wave velocity; TAC: thoracic aortic calcium score; Zc: characteristic impedance of the aorta.

We applied inverse normal transformation to platelet aggregation traits. We then conducted an association analysis between the transformed platelet outcomes and tonometry/calcium/diameter traits using a linear mixed effects model implemented in the R package GMMAT (https://cran.r-project.org/web/packages/GMMAT/index.html) to account for familial correlation. We adjusted for common cardiovascular risk factors, which included height, weight, body mass index, heart rate, fasting plasma total cholesterol, and high-density lipoprotein cholesterol (HDL-C) modeled as the ratio of total to HDL-C, triglycerides (TG), glucose, self-report of smoking within the past year, prevalent diabetes or CVD, and treatment for hypertension or hyperlipidemia. As previously described, independent trait analysis suggests correction for 45 platelet traits.^34^ Here as five principal components explained 90% of trait variation among tonometry, diameter, and calcium traits, we considered *p* < .05/(45 × 5) = 2.22e-04 to be statistically significant in analyses. We describe results with *p* < .05 in a two-sided test as “trends.” Following the analyses, Z-scores were calculated from beta (β) and standard error values (β/SE). To accurately represent the directionality of the associations with regard to increased platelet reactivity, the β and Z-statistics presented in Figure 2, Tables II–IV, and Tables S3-S5 for following variables were transformed by multiplying a factor of −1: Agg20, Agg40, ecMax, EC50, disaggregation traits (4 total), and lag time traits (2 total). Results from the cardiovascular risk factor-adjusted model are shown in Tables II–IV. Results from the age-, sex-, and aspirin use-adjusted model are shown in Tables S3-S5. The independent variables were arterial tonometry, aortic and arterial diameter, and aortic and arterial calcium, while the dependent variables were platelet function measures.

## Results

The top results from our analysis are shown in Tables II–IV. To summarize, all calcium, diameter, and tonometry traits failed to reach a stringent multiple test correction significance threshold (*p* < 2.22e-04). However, numerous traits across all three categories had p-values <0.05. In order to better visualize the results by agonist tests and traits, we created Figure 2; this is a heatmap of the z-statistics of associations between calcium, diameter, tonometry, and platelet activation measures, grouped by independent variable type with p-value trends marked (*p ≤ .05, **p ≤ .01). This plot emphasizes trends between individual platelet activation traits and calcium, diameter, and tonometry traits.

### Arterial calcium and platelet reactivity

Generally, arterial calcium measures showed positive trends with platelet reactivity traits as shown by the greater number of red boxes for calcium traits in Figure 2. The most significant trends were between abdominal aorta calcium scores (AAS) and AA platelet reactivity in Optimul and collagen platelet reactivity in LTA, and ristocetin-induced platelet agglutination in Optimul (*p* = 4.8e-03–0.04) [Figure 2, Table II]. The trends mostly reflected higher abdominal calcium being associated with lower platelet reactivity, except for collagen in the Optimul assay. TAC trended with increased platelet reactivity for TRAP-6 in LTA, U46619 in Optimul, as well as increased ristocetin-induced platelet agglutination in LTA (*p* = .01–0.05) [Figure 2, Table II]. WB ADP reactivity results by flow cytometry trended in a counter direction with TAC (*p* = .01–0.04). For CAC, there were trends with 1.82 μM ADP in LTA (*p* = 7.5e-03–0.04) [Figure 2, Table II]. 0.95 μM ADP in LTA showed similar trends of increased reactivity with higher CAC (*p* = .01–0.04). In addition, higher CAC trended with lower U46619-induced platelet reactivity (*p* = .03) and higher AA-induced reactivity in LTA (*p* = .04).

### Aortic and arterial diameter measures

The majority of aortic and arterial diameter measures had negative trends with platelet reactivity traits as shown by the greater number of blue boxes for diameter traits in Figure 2. Abdominal aorta and ascending, descending thoracic aorta diameters had the most significant negative trends with various epinephrine, ADP, and U46619-induced aggregation, T-TAS collagen-induced thrombus formation, and ristocetin-induced platelet agglutination in LTA and Optimul (*p* = 1.1e-03–0.05) [Figure 2, Table III].

### Arterial tonometry measures and platelet reactivity

There was a combination of positive and negative trends between various tonometry and platelet reactivity traits as shown by the combination of red and blue boxes for tonometry traits in Figure 2. There were positive trends between Zc and Multiplate collagen AUC, LTA collagen primary slope, as well as various AA and epinephrine-induced aggregation traits (*p* = 2.6e-03–0.0498). There was a negative trend between Zc and collagen primary slope (*p* = .01) [Figure 2, Table IV].

In addition, there were negative trends between niCFPWV, 0.95 μM ADP secondary aggregation and secondary slope in LTA and epinephrine ecMax and slope (*p* = 2.4e-03–7.4e-03; *p* = .01–0.03, respectively). There were positive trends between CPP and 0.95 μM ADP primary aggregation and primary slope in LTA (*p* = 2.8e-03–8.5e-03) [Figure 2, Table IV].

## Discussion

We observed a pattern of negative trends for larger measured mean blood vessel diameters with lower platelet activation. Ascending, descending thoracic aorta, and abdominal aorta measures had the most significant trends with ADP in Optimul, epinephrine in Optimul, and shear-mediated collagen-induced thrombus formation, among other platelet reactivity variables. Arterial calcium measures mostly displayed positive trends with platelet reactivity, with AAS having the most significant trends with AA reactivity in LTA, collagen-induced platelet reactivity in LTA, and ristocetin-induced platelet agglutination in Optimul. CAC and response to 1.82 μM ADP in LTA; and TAC, TRAP-6 platelet reactivity in LTA, and U46619-induced platelet reactivity in Optimul also showed positive trends. WB ADP reactivity measured by flow cytometry showed a negative trend with TAC. For the arterial tonometry measures, Zc, and CPP trended positively with multiplate collagen, Optimul epinephrine, LTA ADP, and other platelet reactivity traits. niCFPWV and Zc trended negatively with ADP, collagen, and epinephrine measures.

The vessel diameter results are consistent with the concept of a lower shear stress environment that may provide a milieu of decreased platelet reactivity. Prior research has shown that platelet activation can be provoked by shear forces and potentiated by higher shear stress. For instance, the shear-mediated interaction between GPIbα on the surface of platelets and vWF on the exposed subendothelial extracellular matrix that is exposed at sites of vascular injury initiates a signaling cascade that results in platelet adhesion, activation, and thrombus formation.^43–45^ In addition to yield stress and the other non-Newtonian rheological behaviors of blood, this action can promote thrombosis and pathological blockages seen in CVDs such as strokes.^46^

Second, the pattern of our nominally associated calcium results is consistent with other evidence suggesting that subclinical atherosclerosis and calcium buildup may provoke platelet activation through various pathways, contributing to higher thrombosis risk. For example, Chirumamilla et al. studied patients who were undergoing percutaneous coronary intervention and were receiving clopidogrel therapy; high on-treatment platelet reactivity was associated with longer atherosclerotic lesions with greater volumetric dimensions.^47^ Various mechanisms have been proposed to explain the relationship between atherosclerotic burden and platelet reactivity, such as platelet activating factor (PAF) activating platelets and leukocytes, enhancing leukocyte adhesion to the vessel wall, and by extension, plaque formation.^48,49^ Also, Marx et al. illustrated that eosinophils support atherosclerotic plaque formation by facilitating endothelial cell vWF exposure, which promoted greater platelet adhesion.^50^ Furthermore, it is known that atherosclerotic plaque rupture results in the exposure of the procoagulant subendothelial matrix, which can promote platelet and leukocyte recruitment and activation, leading to pathogenic arterial thrombosis.^51^ Prior work in the FHS showed that ADP platelet hyperreactivity was associated with future CVD after accounting for traditional CVD risk factors.^4^ Likewise, Berger et al.’s study in the LURIC cohort indicated higher ADP platelet reactivity may be an independent predictor of future CVD mortality.^1^ The observed positive trends between higher concentration (1.82 μM) ADP platelet activation and coronary artery calcium levels suggests a connection between atherosclerotic burden and ADP platelet activation pathways, and is consistent with platelet P2Y12 inhibitor therapy in CVD prevention.

It is known that activated platelets and endothelial cells recruit other immune cells, such as monocytes and neutrophils, promoting thrombus stabilization by delivering tissue factor and neutrophil extracellular trap formation.^52,53^ Increased calcium area and volume are associated with increased atherosclerotic plaque area and volume,^16,54^ and calcified plaque is also a well-established marker for adverse cardiovascular events and atherosclerotic progression since it is strongly associated with total plaque burden.^54^ Furthermore, a high CAC burden has been associated with atrial fibrillation, extra coronary atherosclerosis, increased risk of cerebrovascular events, and an unfavorable CVD risk profile.^55–57^ Our results both reinforce these previous findings and point to a relationship between increased vessel calcium burden, platelet activation, and higher thrombotic and atherosclerotic risk that should be investigated further. However, these results do not establish a causal direction for such trends.

The tonometry results are consistent with a possible diameter-mediated effect on Zc and niCFPWV, even though the link with platelet activation did not reach a stringent multiple testing significance threshold. It is known that increased arterial stiffness, measured by pulse wave velocity, is an independent risk factor of atherosclerotic CVD,^58^ and in atherosclerotic disease patients, platelets are likely to be activated.^59^ In addition, Zc and CFPWV were found to be correlated to each other (*p* < 10^−4^) with confounders, and both were independently associated with carotid intima-media thickness and endothelial activation markers.^60^ Both carotid intima- media thickness and carotid artery measures appear to be correlated with one another, and both have been used as predictors of cardiovascular events in large population studies.^28,61^ From this, there may be a mechanistic relationship between arterial tonometry measures and platelet activation measures that should be elucidated through further research using larger tonometry measure samples gathered from diverse cohorts.

One limitation of this study was the limited amount of tonometry and platelet function data available from the older age NOS and the ethnically diverse OMNI-2 cohort. This may affect the generalizability of the results to the U.S. population, and is a problem shared with most other population studies of platelet function, indicating an important issue to address in future studies. A second potential limitation is the inability to replicate these findings in other cohorts, as there have been few population studies that have gathered platelet function, arterial tonometry, and vascular function data to the extent of FHS. However, this reflects the novelty of our study because, to our knowledge, there have been very few age-, ethnically-and-racially-diverse community-based studies to date that have gathered data on and assessed associations between platelet function, tonometry, arterial and aortic diameter, and arterial calcium measures to the extent of FHS. Most other cardiovascular epidemiological studies have either occurred over shorter periods, have not assessed one of the four aforementioned measures, or have had smaller sample sizes compared to FHS.^62–65^ A third potential limitation is the time between the aortic and arterial diameter and calcium measures. Since approximately 14 years elapsed between Exams 1 and 3, the time gap could bias the results toward a null association if no or weak relations are observed between variables. Conversely, true associations may be stronger if measured concurrently. However, prior cross-sectional studies of different vessel anatomy and scanning techniques from young adulthood to later age in thousands of individuals^66–68^ have all suggested that there is a fairly linear increase in vessel diameters throughout these decades across men, women and ancestry groups. Additionally, vessel calcium measures are thought to change slowly in most adults. Previous cross-sectional studies using various scanning techniques and measuring different vessel calcium variables in thousands of individual across a large range of ages^69,70^ indicate that vessel calcium burden likely shows relatively steady increases throughout adulthood until advanced age, where there may be a more rapid acceleration of calcium burden. Since the time periods covered in our subjects are from early-middle age to later-middle age, we believe the cohort generally would have been in a phase of linear growth in these measurements. Furthermore, after adjusting for the number of years between imaging and platelet function measurements, the trends did not change appreciably and showed high concordance of effect (calcium traits *r* = 0.999, diameter traits *r* = 0.919). A fourth limitation is that all the results did not meet a more stringent multiple testing threshold (*p* < 2.22e-04) and only had suggestive trends (*p* < .05). Unfortunately, since there are no cohort studies that have gathered similar amounts of tonometry, platelet activation, and calcium measures as FHS; we cannot replicate these results to see if there are more significant relationships. However, our results are consistent with prior research on individual associations between these various measures. A final limitation is that due to the association-based analyses that form the basis of this study, we cannot determine the causal direction for the observed relationships. In the future, it could be worthwhile to apply causal inference methods like Mendelian randomization to genetic data, as there are increasingly well-powered studies for vessel diameters,^29^ calcium,^71,72^ tonometry and vessel stiffness measures,^73–75^ platelet activation,^76,77^ and CVD outcomes with regard to known risk factors.^78–80^

To date, this is the largest community-based cohort study to examine the relationships between platelet function, arterial tonometry, aortic and arterial diameter, and calcium arterial bed burden measures. Our exploratory findings should inform further work designed to evaluate various associations between the same or other tonometry and platelet activation measures. More direct measures of endothelial and microvascular function were not assessed in this study. In a prior study, flow-mediated dilation (FMD) was found to be reduced in a sample of 28 unprovoked venous thromboembolism (VTE) patients when compared to non-VTE referents, as well as increased levels of vWF and soluble P-selectin in plasma, both of which are platelet and endothelial activation markers.^81^ In another study that focused on lone atrial fibrillation parameters, FMD, and diameter measures, FMD was found to be significantly reduced and the lone atrial fibrillation and diameter measures were slightly increased in a sample of 43 lone atrial fibrillation patients.^82^ From both studies, it is clear that significantly hindered vessel function measures are associated with cardiovascular outcomes. Future investigation using additional data being collected in FHS on tonometry, endothelial and microvascular and platelet activation datasets could yield more insight into the connection between those variables and cardiovascular outcomes.

## Supplementary Material

Supp 1

Supp 2

## Figures and Tables

**Table I.** Demographic information.

	Tonometry (2016–2019)	Calcium (2008–2011)	Diameter (2002–2005)
Trait	Mean (SD)/N(%)	Mean (SD)/N(%)	Mean (SD)/N(%)
Sex (female)	1807 (53.88)	762 (45.71)	763 (46.10)
Age (years)	54.40 (9.27)	58.84 (5.94)	58.88 (5.98)
Lipid Medications	836 (24.93)	546 (32.77)	544 (32.89)
Anti-hypertensive medications	934 (27.86)	577 (34.63)	572 (34.58)
Aspirin use	716 (21.35)	460 (27.59)	456 (27.55)
P2Y12 inhibitor use	32 (0.95)	19 (1.14)	22 (1.33)
BMI (kg/m2)	28.49 (5.77)	28.93 (5.79)	28.93 (5.80)
Total Cholesterol (mg/dL)	189.39 (36.60)	190.70 (36.70)	190.73 (36.89)
HDL (mg/dL)	59.58 (19.47)	59.31 (20.41)	59.37 (20.52)
Fasting glucose (mg/dL)	100.10 (21.18)	102.65 (23.12)	102.58 (22.99)
Smoking	217 (6.47)	97 (5.82)	99 (5.99)
Type 2 diabetes	296 (8.83)	186 (11.16)	185 (11.19)
AAo (*N* = 1649)			31.94 (3.60)
DAo (*N* = 1649)			23.39 (2.43)
AbAo (*N* = 1655)			17.33 (1.88)
CAC (*N* = 1667)		467 (28.01)	
TAC (*N* = 1415)		483 (34.13)	
AAS (*N* = 1421)		658 (46.31)	
Zc (*N* = 3354) (dyne × sec/cm^5^	210.20 (74.56)		
CPP(*N* = 3354) (mmHg)	60.04 (16.34)		
niCFPWV (*N* = 3349) (ms/m)	−130.65 (26.41)		

The following table is a summary of the relevant demographic, tonometry, diameter, and calcium measures gathered from Gen3/NOS/OMNI-2 cohorts. Platelet and tonometry measures were gathered from Gen3/NOS/OMNI-2 cohorts during Exam 3 (2016–2019), vessel calcium measures were gathered from Gen3/OMNI-2 cohorts during Exam 2 (2008–2011), and vessel diameter measures were gathered from Gen3 cohort during Exam 1 (2002–2005). *N* = 3,429.

Abbreviations: AAo: antero-posterior diameter of the ascending thoracic aorta obtained at the level of the right pulmonary artery; DAo: Antero-posterior diameter of the descending thoracic aorta obtained at the level of the right pulmonary artery; AbAo: antero-posterior diameter of the abdominal aorta 5 cm above the aorto-common iliac bifurcation; CAC: coronary artery calcium score; TAC: thoracic aortic calcium score; AAS: abdominal aorta calcium score; Zc: characteristic impedance of the aorta; CPP: central pulse pressure; niCFPWV: negative inverse carotid femoral pulse wave velocity.

**Table II.** Aortic and arterial calcium results (risk factor-adjusted model).

Agonist	Concentration	Assay Type	Platelet Trait	Calcium Trait	Beta	SE	Z-Statistic	P-Value	N
AA	0.03–1.0 mM	Optimul	ecMAX	AAS	−0.147	0.052	−2.824	4.75E–03	1162
ADP	1.82 μM	LTA	FinAgg	CAC	0.143	0.053	2.672	7.54E–03	1586
ADP	20 μM	Flow Cytometry (WB)	PAC1% (WB)	TAC	−0.18	0.071	−2.537	1.12E–02	1153
Ristocetin	1.5 mg/mL	LTA	FinAgg	TAC	0.168	0.066	2.528	1.15E–02	1351
ADP	0.95 μM	LTA	PrimSlope	CAC	0.143	0.057	2.498	1.25E–02	1502
Ristocetin	1.5 mg/mL	LTA	MaxAgg	TAC	0.162	0.066	2.457	1.40E–02	1351
ADP	1.82 μM	LTA	MaxAgg	CAC	0.133	0.056	2.395	1.66E–02	1586
Ristocetin	1.5 mg/mL	LTA	AUC	TAC	0.158	0.067	2.364	1.81E–02	1350
ADP	1.82 μM	LTA	AUC	CAC	0.126	0.054	2.330	1.98E–02	1586
Collagen	190 μg/mL	LTA	PrimSlope	AAS	−0.119	0.052	−2.314	2.07E–02	1370
Ristocetin	0.14–4.0 mg/ml	Optimul	ecMAX	AAS	−0.122	0.053	−2.289	2.21E–02	1234
ADP	1.82 μM	LTA	PrimSlope	CAC	0.126	0.058	2.178	2.94E–02	1586
U46619	0.005–40 μM	Optimul	ecMAX	CAC	−0.121	0.056	−2.174	2.97E–02	1442
Collagen	0.01–40 μg/ml	Optimul	ecMAX	AAS	0.09	0.043	2.098	3.59E–02	1256
Collagen	190 μg/mL	LTA	AUC	TAC	0.113	0.054	2.097	3.60E–02	1364
U46619	0.005–40 μM	Optimul	eMax	TAC	0.146	0.07	2.097	3.60E–02	1243
Ristocetin	1.5 mg/mL	LTA	PrimSlope	TAC	0.134	0.065	2.069	3.85E–02	1350
ADP	20 μM	Flow Cytometry (WB)	PAC1_Psel_DoublePos% (WB)	TAC	−0.147	0.071	−2.067	3.88E–02	1153
TRAP-6	0.67 mM	LTA	PrimAgg	TAC	0.135	0.066	2.046	4.07E–02	1360
AA	1.6 mM	LTA	FinAgg	CAC	0.087	0.043	2.038	4.16E–02	1587
ADP	0.95 μM	LTA	MaxAgg	CAC	0.118	0.058	2.029	4.25E–02	1502
TRAP-6	0.67 mM	LTA	PrimSlope	TAC	0.134	0.068	1.976	4.81E–02	1360

Trending results (*p* < .05) of multiple testing analysis of aortic and arterial calcium traits and platelet traits. Positive beta/z-statistic values indicate increased platelet reactivity with an increase in the cardiovascular traits; negative beta/z-statistic values indicate reduced platelet reactivity with a decrease in the cardiovascular traits. We used linear mixed effects models, which included CVD risk factors, to calculate the betas (β) and standard errors (SE) for this display. Calcium values had 1 added and were ln-transformed. Following this transformation, the β and SE values were used to calculate z-statistics (β/SE). The following z-statistic values were multiplied by −1 to accurately depict the platelet activation directionality: Agg20, Agg40, ecMax, EC50, disaggregation traits (4 total), and lag time traits (2 total).

Abbreviations: CAC: coronary artery calcium score; AAS: abdominal aorta calcium score; TAC: thoracic aorta calcium score.

**Table III.** Vessel diameter results (risk factor-adjusted model).

Agonist	Concentration	Assay Type	Platelet Trait	Diameter Trait	Beta	SE	Z-Statistic	P-Value	N
Epinephrine	0.0004–10 μM	Optimul	eMax	DAo	−0.048	0.015	−3.251	1.15E–03	1468
ADP	0.005–40 μM	Optimul	eMax	DAo	−0.046	0.016	−2.949	3.18E–03	1473
Epinephrine	0.0004–10 μM	Optimul	eMax	AbAo	−0.053	0.019	−2.820	4.81E–03	1472
TRAP-6	1.5 μM-1.8 mM	Optimul	eMax	DAo	−0.045	0.016	−2.735	6.24E–03	1284
Ristocetin	1.5 mg/mL	LTA	PrimAgg	AAo	−0.019	0.008	−2.311	2.08E–02	1557
AA	0.03–1.0 mM	Optimul	Slope	DAo	−0.037	0.016	−2.285	2.23E–02	1329
TRAP-6	1.5 μM-1.8 mM	Optimul	AUC	DAo	−0.038	0.017	−2.275	2.29E–02	1284
AA	0.03–1.0 mM	Optimul	ecMAX	DAo	−0.029	0.013	−2.266	2.35E–02	1330
Collagen (shear)	N/A	T-TAS	AUC	DAo	0.059	0.026	2.246	2.47E–02	399
Ristocetin	0.14–4.0 mg/ml	Optimul	EC50	AAo	−0.019	0.009	−2.243	2.49E–02	1414
Ristocetin	0.14–4.0 mg/ml	Optimul	Agg40	AAo	−0.019	0.009	−2.199	2.79E–02	1404
ADP	0.005–40 μM	Optimul	Agg40	DAo	−0.03	0.014	−2.176	2.96E–02	1457
TRAP-6	1.5 μM-1.8 mM	Optimul	eMax	AbAo	−0.045	0.021	−2.139	3.24E–02	1286
ADP	5.71 μM	LTA	AUC	AbAo	−0.035	0.017	−2.104	3.54E–02	1566
U46619	0.005–40 μM	Optimul	AUC	DAo	−0.032	0.015	−2.093	3.63E–02	1430
U46619	0.005–40 μM	Optimul	Agg40	DAo	−0.032	0.015	−2.085	3.71E–02	1411
Ristocetin	1.5 mg/mL	LTA	Disagg	AbAo	0.03	0.015	2.051	4.02E–02	1563
Ristocetin	0.14–4 mg/ml	Optimul	eMax	DAo	−0.032	0.016	−2.044	4.10E–02	1446
ADP	5.71 μM	LTA	MaxAgg	AbAo	−0.033	0.016	−2.020	4.34E–02	1568
U46619	0.005–40 μM	Optimul	EC50	DAo	−0.03	0.015	−2.008	4.46E–02	1417
Ristocetin	1.5 mg/mL	LTA	PrimSlope	AAo	−0.017	0.008	−2.004	4.51E–02	1557
Epinephrine	100 μM	LTA	PrimSlope	AAo	−0.015	0.007	−1.985	4.71E–02	1570
TRAP-6	1.5 μM-1.8 mM	Optimul	aucMean	AbAo	−0.042	0.021	−1.971	4.87E–02	1286
U46619	0.005–40 μM	Optimul	ecMAX	DAo	−0.026	0.013	−1.970	4.88E–02	1430
ADP	0.005–40 μM	Optimul	aucMean	DAo	−0.028	0.014	−1.967	4.92E–02	1471
Epinephrine	0.0004–10 μM	Optimul	aucMean	DAo	−0.028	0.014	−1.963	4.96E–02	1462
ADP	0.005–40 μM	Optimul	EC50	DAo	−0.027	0.014	−1.963	4.97E–02	1461

Trending results (*p* < .05) of multiple testing analysis of aortic and arterial diameter traits and platelet traits. Positive beta/z-statistic values indicate increased platelet reactivity with an increase in the cardiovascular traits; negative beta/z-statistic values indicate reduced platelet reactivity with a decrease in the cardiovascular traits. We used linear mixed effects models, which included CVD risk factors, to calculate the betas (β) and standard errors (SE) for this display. The β and SE values were used to calculate z-statistics (β/SE). The following z-statistic values were multiplied by −1 to accurately depict the platelet activation directionality: Agg20, Agg40, ecMax, EC50, disaggregation traits (4 total), and lag time traits (2 total).

Abbreviations: AAo: antero-posterior diameter of the ascending thoracic aorta obtained at the level of the right pulmonary artery; AbAo, antero- posterior diameter of the abdominal aorta 5 cm above the aorto-common iliac bifurcation; DAo, Antero-posterior diameter of the descending thoracic aorta obtained at the level of the right.

**Table IV.** Arterial tonometry analysis results (risk factor-adjusted model).

Agonist	Concentration	Assay Type	Platelet Trait	Tonometry Trait	Beta	SE	Z-Statistic	P-Value	N
ADP	0.95 μM	LTA	SecSlp	niCFPWV	−0.0019	0.00064	−3.040	2.36E–03	3049
Collagen	61.0 μg/mL	Multiplate	AUC	Zc	0.00072	0.00024	3.010	2.59E–03	3336
ADP	0.95 μM	LTA	PrimAgg	CPP	0.0034	0.0011	2.990	2.81E–03	3059
ADP	0.95 μM	LTA	SecAgg	niCFPWV	−0.0017	0.00064	−2.680	7.39E–03	3049
ADP	0.95 μM	LTA	PrimSlope	CPP	0.0029	0.0011	2.630	8.46E–03	3059
Epinephrine	0.0004–10 μM	Optimul	ecMax	niCFPWV	−0.0018	0.00071	−2.548	1.08E–02	2987
Ristocetin	1.5 mg/mL	LTA	PrimSlope	CPP	−0.0029	0.0012	−2.490	1.26E–02	3224
Collagen	190 μg/mL	LTA	PrimSlope	Zc	−0.00054	0.00022	−2.460	1.40E–02	3264
Epinephrine	0.0004–10 μM	Optimul	aucMean	Zc	0.00060	0.00025	2.370	1.78E–02	2996
Epinephrine	0.0004–10 μM	Optimul	eMax	Zc	0.00058	0.00026	2.260	2.41E–02	3014
Epinephrine	0.0004–10 μM	Optimul	Slope	niCFPWV	−0.0021	0.00094	−2.200	2.80E–02	2986
Ristocetin	1.5 mg/mL	LTA	FinAgg	niCFPWV	0.0020	0.00090	2.200	2.80E–02	3212
Ristocetin	1.5 mg/mL	LTA	MaxAgg	niCFPWV	0.0019	0.00090	2.130	3.34E–02	3212
Epinephrine	100 μM	LTA	SecAgg	niCFPWV	0.0015	0.00070	2.090	3.67E–02	3250
Epinephrine	100 μM	LTA	SecSlp	niCFPWV	0.0014	0.00070	2.030	4.19E–02	3250
AA	0.03–1.0 mM	Optimul	Agg40	Zc	0.00046	0.00023	2.027	4.27E–02	2658
ADP	0.95 μM	LTA	PrimAgg	Zc	0.00051	0.00025	2.020	4.36E–02	3059
Collagen	61.0 μg/mL	Multiplate	Agg	Zc	0.00048	0.00024	1.980	4.76E–02	3336
Collagen	61.0 μg/mL	Multiplate	AUC	niCFPWV	−0.0017	0.00085	−1.960	4.96E–02	3324
Epinephrine	0.0004–10 μM	Optimul	Agg20	Zc	0.00052	0.00027	1.962	4.98E–02	2943

Trending results (*p* < .05) of multiple testing analysis of arterial tonometry and platelet traits. Positive beta/z-statistic values indicate increased platelet reactivity with an increase in the cardiovascular traits; negative beta/z-statistic values indicate reduced platelet reactivity with a decrease in the cardiovascular traits. We used linear mixed effects models, which included CVD risk factors, to calculate the betas (β) and standard errors (SE) for this display. The β and SE values were used to calculate z-statistics (β/SE). The following z-statistic values were multiplied by −1 to accurately depict the platelet activation directionality: Agg20, Agg40, ecMax, EC50, disaggregation traits (4 total), and lag time traits (2 total).

Abbreviations: niCFPWV: negative inverse carotid femoral pulse wave velocity; CPP: central pulse pressure; Zc: characteristic impedance of the aorta.

## Data Availability

Framingham Heart Study variables are deposited in the NIH dbGaP repository under the accession # phs000007.v32.p13 and available for application to access by qualified researchers https://www.ncbi.nlm.nih.gov/gap/.

## Plain lanugage summary

### What is the context?

- Prior research has reported that measures of vascular system-influencing proteins such as angiopoietin-2, arterial calcium plaque formation, and arterial stiffness assessed by tonometry are associated with CVD risk.
- Since activated platelets produce and release vascular proteins like angiopoietin when activated, and microparticles that interact with endothelium, release of the foregoing mediators could provide one way in which vascular structure and platelet function influence each other.
- To our knowledge, no prior studies have directly investigated associations between these measures in a large sample. This investigation relates platelet function to arterial tonometry, aortic and arterial diameter, and arterial calcium measures in the Framingham Heart Study (FHS) Gen3/NOS/OMNI-2 cohorts (*n* = 3,429).

### What’s new?

- Generally, higher arterial calcium measures trended with higher platelet reactivity, whereas larger aortic diameters trended with lower platelet reactivity.
- Arterial tonometry measures had positive and negative trends with platelet functions, including platelet measures with opposite relations to negative-inverse carotid-femoral pulse wave velocity (niCFPWV) and characteristic impedance (Zc). All tonometry, calcium, and diameter results did not reach a more stringent multiple testing threshold (*p* < 2.22e-04).

### What’s the impact?

- The aortic diameter trends are consistent with lower shear stress invoking less platelet reactivity.
- The vessel calcium trends are consistent with increased vascular calcium buildup that could provoke platelet activation, thereby contributing to increased blood clot risk. Conversely, increased platelet activation could contribute to increased inflammation and thrombosis, leading to calcification in the arterial wall.

## Abbreviations

AA: arachidonic acid
AAo: antero-posterior diameter of the ascending thoracic aorta obtained at the level of the right pulmonary artery
AbAo: antero-posterior diameter of the abdominal aorta 5 cm above the aorto-common iliac bifurcation
AAS: abdominal aorta calcium score
ADP: adenosine diphosphate
Agg20: agonist concentration needed to reach 20% aggregation
Agg40: agonist concentration needed to reach 40% aggregation
AUC: area-under-the-curve
aucMean: AUC across concentration range
BMI: body mass index
CAC: coronary artery calcium score
CT: computed tomography
CPP: central pulse pressure
CVD: cardiovascular disease
DAo: Antero-posterior diameter of the descending thoracic aorta obtained at the level of the right pulmonary artery
ecMax: agonist concentration needed to reach maximal aggregation
eMax: maximal % aggregation (Optimul)
EC50: agonist concentration needed to reach 50% aggregation
FHS: Framingham Heart Study
FinAgg: final % aggregation
FMD: flow-mediated dilation
GP: glycoprotein
HDL-C: high-density lipoprotein cholesterol
LTA: light transmission aggregometry assay
LURIC: LUdwigshafen RIsk and Cardiovascular Health Study
n/a: information not available
niCFPWV: negative inverse carotid femoral pulse wave velocity
NOS: New Offspring Spouse
NSAID: non-steroidal anti-inflammatory drug
MaxAgg: maximal % aggregation
PAC1_Psel_DoublePos%: Percent PAC1+ and CD62P+ positive platelets
PAC1%: Percent PAC1+ positive platelets
PrimSlope: primary wave slope
PRP: platelet-rich plasma
SecAgg: secondary wave % aggregation
SecSlp: secondary wave slope
Slope: slope across concentration range (Optimul)
TAC: thoracic aortic calcium score
TG: triglycerides
T-TAS: total-thrombus formation assay system
TRAP-6: thrombin receptor activator peptide-6
vWF: von Willebrand Factor
VTE: venous thromboembolism
WB: whole blood
Zc: characteristic impedance of the aorta
