# Supplementary figures and images for: Trends among platelet function, arterial calcium, and vascular function measures
Source: Platelets. Author manuscript; Available in PMC 2024 Mar 18. (PMC10947606; doi:10.1080/09537104.2023.2238835)

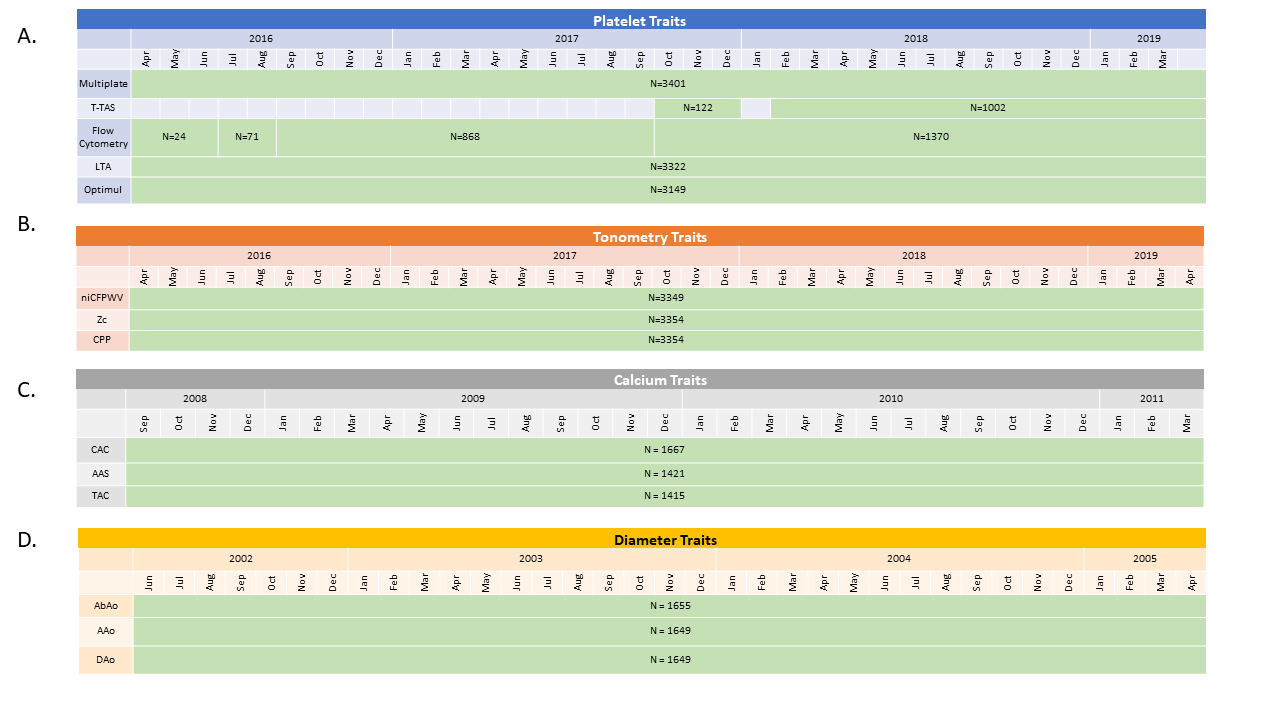

Supplement: Supp 1 [file NIHMS1923445-supplement-Supp_1.tif]
